# Mechanistic modelling of within-mosquito viral dynamics: Insights into infection and dissemination patterns

**DOI:** 10.1371/journal.pcbi.1011520

**Published:** 2023-10-09

**Authors:** Jennifer S. Lord, Michael B. Bonsall

**Affiliations:** 1 Department of Vector Biology, Liverpool School of Tropical Medicine, Liverpool, United Kingdom; 2 Department of Biology, University of Oxford, Oxford, United Kingdom; Utrecht University, NETHERLANDS

## Abstract

Vector or host competence can be defined as the ability of an individual to become infected and subsequently transmit a pathogen. Assays to measure competence play a key part in the assessment of the factors affecting mosquito-borne virus transmission and of potential pathogen-blocking control tools for these viruses. For mosquitoes, competence for arboviruses can be measured experimentally and results are usually analysed using standard statistical approaches. Here we develop a mechanistic approach to studying within-mosquito virus dynamics that occur during vector competence experiments. We begin by developing a deterministic model of virus replication in the mosquito midgut and subsequent escape and replication in the hemocoel. We then extend this to a stochastic model to capture the between-individual variation observed in vector competence experiments. We show that the dose-response of the probability of mosquito midgut infection and variation in the dissemination rate can be explained by stochastic processes generated from a small founding population of virions, caused by a relatively low rate of virion infection of susceptible cells. We also show that comparing treatments or species in competence experiments by fitting mechanistic models could provide further insight into potential differences. Generally, our work adds to the growing body of literature emphasizing the importance of intrinsic stochasticity in biological systems.

## Author summary

Mosquitoes are vectors of viruses, like dengue or Zika, that can cause disease in humans. However, not all mosquito species and not all individuals are equally likely to become infected and subsequently transmit virus. To study the virus infection process in mosquitoes, experiments are usually designed where groups of mosquitoes are provided with an infected blood meal and after an incubation period the proportion of flies that are infected and the proportion that are capable of transmission are assessed. Researchers have shown, using experimental infection, that the amount of virus in a blood meal affects the probability of infection and how long it takes for a mosquito to be able to transmit. Here, we develop a mechanistic model of this process which demonstrates that these phenomena can be explained by stochasticity that is intrinsic to the system.

## Introduction

Mosquito-borne viruses, particularly those transmitted by *Aedes* mosquitoes, are a major global health problem. In 2017, the combined burden of dengue, yellow fever and Japanese encephalitis was estimated to be greater than 2,000,000 disability-adjusted life years (DALYs) [1]. Similarly, between 2010 and 2019, Zika (ZIKV) and chikungunya viruses caused an estimated annual combined loss of greater than 150,000 DALYs [2]. Interventions to reduce the public health burden of these diseases have focused mainly on mosquito control. Control programmes against mosquito-borne viruses often focus on increasing vector mortality to reduce transmission and therefore disease risk. However, any interventions that involve mosquito population suppression, including insecticide-treated bed nets or genetic control, face the problem of resistance evolution in affected populations [3]. The importance of new, and combined, approaches to reducing the burden of mosquito-borne viral disease has therefore been highlighted [4, 5].

Some alternative approaches to control mosquito-borne viruses aim to reduce vector competence. Here, we define vector competence as the ability of mosquitoes to become infected and subsequently transmit virus. Under this definition, the extrinsic incubation period (EIP), which is the time it takes for virus to reach the salivary glands of an infected mosquito, is included as a component of competence [5–7]. At the population-level, vector competence contributes to the force of infection from host-to-mosquito via the probability of mosquito midgut infection given a bite on an infected host. It also influences mosquito-to-host transmission, through the EIP. Approaches that aim to reduce vector competence have gained traction since the successful deployment of *Wolbachia*-infected *Aedes* mosquitoes, rendering them unable to transmit dengue virus. In Yogyakarta, Indonesia, releases of *Wolbachia*-infected *Aedes* reduced dengue incidence by *c*. 70% [8].

Measuring vector competence under experimental conditions plays a key part in the development and assessment of pathogen-blocking control tools. Vector competence experiments are also important more generally in implicating mosquito species in arbovirus transmission [9, 10]. In addition to inter-species differences due to vector and virus genetics, studies have shown that vector competence varies within a species (e.g. [11, 12]), due to both intrinsic and extrinsic factors, including mosquito genetics, microbiome [13], age [14], larval environment and temperature [15]. Variation in these factors, therefore, likely contribute to spatiotemporal variation in outbreak risk [16].

To our knowledge there are no mechanistic models of within-mosquito virus dynamics and vector competence. Vector competence is determined by the ability of virus particles (virions) to infect a mosquito’s midgut epithelial cells, multiply, and escape the midgut to disseminate to other tissues and infect the salivary glands, where they can be transmitted to a vertebrate host [17]. Therefore, to measure vector competence, typically: i) female mosquitoes are exposed to virus via an infected host or artificial blood meal; ii) then these infected mosquitoes are incubated over a pre-specified number of days; after which iii) *in vivo* assessment of virus transmission and/ or assays are performed to detect the presence of virus in the midgut (to demonstrate infection), legs (to demonstrate dissemination), and salivary glands or saliva (to demonstrate an ability to transmit). The resulting experimental data may also be used to estimate the EIP [18]. Data from vector competence experiments, to our knowledge, are usually analysed using separate phenomenological models of infection, dissemination and transmission. Analyses include using ANOVA at single virus concentrations and time points to compare between species or treatments (e.g. [19]) and the calculation of the infectious dose at which 50% of mosquitoes are infected (e.g. [20]). Logistic regression is used to model the probability of infection, dissemination or transmission as a function of virus dose or time (e.g. [21, 22]). Survival analyses of the time response of dissemination and transmission have also been used [23]. Such analyses may give biologically meaningful parameter estimates, but they do not link the initial input virus, the process of midgut infection dynamics with dissemination and transmission. Although, in their study of ZIKV infection in *Ae. albopictus*, Lequime *et. al* mechanistically model virus dynamics in the vertebrate host, their approach to then using this to predict vector competence is phenomenological [22]. Here we show that developing mechanistic models, that explicitly account for viral replication dynamics, would provide a formal definition of vector competence, could inform the design and interpretation of experiments and potentially explain phenomena observed in these empirical investigations.

An important observation, likely common to all mosquito-borne viruses, is that the probability of mosquito midgut infection increases sigmoidally with virus concentration of the blood meal. This has been shown for Barmah forest virus [20], dengue virus [24], Ross River virus [19], Rift Valley fever virus [25], West Nile virus [26] and Zika virus [22] and summarised in [27]. Furthermore, virus concentration in the blood meal not only affects the probability of midgut infection but also the time it takes for virus to disseminate to other tissues, including the salivary glands and thus the EIP [21, 22]. Understanding these processes can inform the design of vector competence experiments comparing different treatments or species.

Despite the possibility of thousands of virions being present in a blood meal, infection in the midgut is typically initiated in very few (*c*. < 15) cells [28–30]. Given this small founding population, the dose-response phenomena of midgut infection and between-individual variation in the duration of the EIP suggest that infection could arise from stochastic processes in the mosquito. Lord (2006) [31] show that infection of vectors should be considered in a probabilistic sense, however their work focused only on the process of midgut infection. In addition, their probabilistic model of mosquito midgut infection focused only on modelling a random distribution of virions in the blood, and they assumed that a threshold level of virions was still required for midgut infection.

Here we present a mechanistic model of virus dynamics within the mosquito, focusing on midgut infection and dissemination to the hemocoel. We aimed to: i) show that the dose-response of the probability of mosquito midgut infection and the effect of virus concentration in the blood meal on dissemination patterns is an emergent property of the stochastic system; and ii) demonstrate that the mechanistic model can produce results similar to that observed in experimental tests of vector competence. Our model provides a complementary approach that could be used alongside phenomenological models to provide further mechanistic insight.

## Materials and methods

### Deterministic model

We model virus infection of cells in the mosquito midgut and hemocoel using a set of coupled ordinary differential equations ODEs (Eq 1), which track the numbers of free virions in the blood meal (*G*_*v*_), the number of infected midgut cells (*M*_*c*_), the number of virions within the midgut epithelium (*M*_*v*_) and in the hemocoel (*H*_*v*_) and the number of infected cells in the hemocoel (*H*_*c*_):

$$\begin{array}{ccc} \frac{dG_{v}}{dt} & = & -G_{v}\beta \left(c_{max}-M_{c}\right)-\mu_{v}G_{v}\\ \frac{dM_{c}}{dt} & = & G_{v}\beta \left(c_{max}-M_{c}\right)+\alpha M_{c}\left(c_{max}-M_{c}\right)\\ \frac{dM_{v}}{dt} & = & \gamma M_{c}-\mu_{v}M_{v}-\rho M_{v}\\ \frac{dH_{v}}{dt} & = & \rho M_{v}+\gamma H_{c}-\mu_{v}H_{v}\\ \frac{dH_{c}}{dt} & = & H_{v}\beta \left(h_{max}-H_{c}\right) \end{array}$$ (1)

where *β* is the rate at which virions infect susceptible cells (combining the contact rate and the probability of infection), *c*_*max*_ and *h*_*max*_ are the total number of cells in the midgut and hemocoel respectively, *μ*_*v*_ is the virus decay rate, *γ* is the rate at which virions are produced by infected cells, *α* is the rate at which virions spread between infected and susceptible midgut cells, and *ρ* is the rate at which virions escape from the midgut epithelium into the hemocoel.

**Fig 1:**
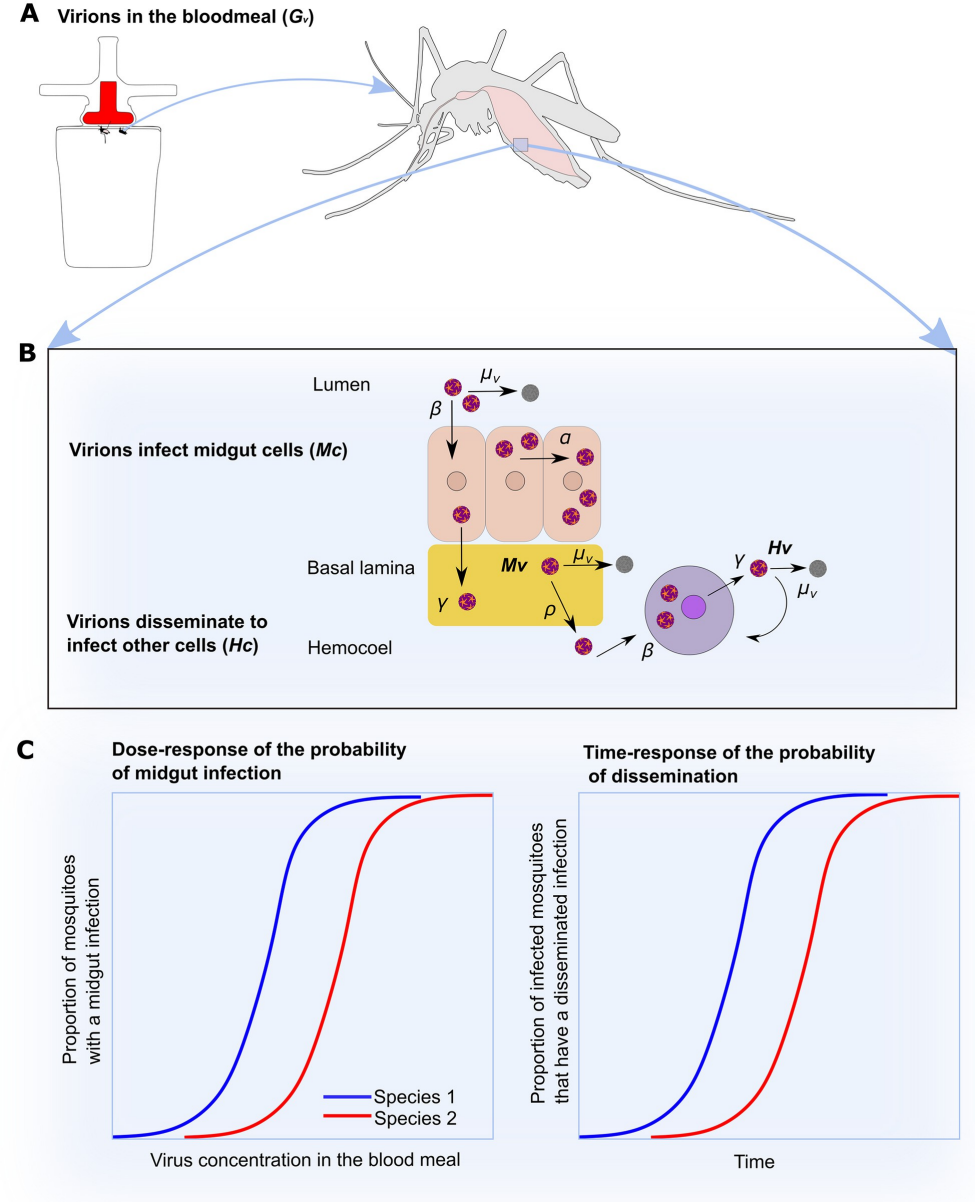
Model schematic and relation to results from vector competence experiments. A: In a vector competence experiment, mosquitoes are provided a blood meal containing virus. Individual mosquitoes ingest *G*_*v*_ virions. After virions enter the midgut, infection must be initiated as shown in B. B: Ingested virions infect susceptible midgut epithelial cells (*M*_*c*_) at rate *β* and can spread between cells at rate *α*. New virions are produced at rate *γ* to produce free virus *M*_*v*_ and can escape through the basal lamina at rate (*ρ*). Here only infected hemocoel cells are modelled (*H*_*c*_) to represent dissemination and free virus in the hemocoel (*H*_*v*_). Virus is cleared at all stages at rate *μ*_*v*_. We also make the simplifying assumption that parameter values are the same across tissues. C: Vector competence experiments usually compare at least two treatments or mosquito species to quantify the ability of mosquitoes, under set experimental conditions to become infected by and subsequently transmit virus; here shown as conceptual results for two different mosquito species. In simulations of the stochastic model and as realised in experiments, multiple individuals can be tracked and the proportion which develop a midgut infection quantified in addition to the proportion infected which develop a disseminated infection. Here we argue that these processes should be seen as functions of initial number of input virions in the blood meal and time post blood meal respectively.

We make the assumption that there is no cell death or virus-induced apoptosis; virions escape cells via budding from the cell membrane. While this assumption likely doesn’t hold in nature [32], relaxing this simplification will be dealt with in future work. We also assume that the rate at which susceptible cells become infected and the rate at which infected cells produce virions are the same for both cells in the midgut and hemocoel. Again this is a simplification of what is likely to occur. The main drivers of viral decay in the midgut will be blood meal digestion together with the formation of the peritrophic matrix [33] and in the hemocoel will be natural decay or clearance due to immune responses [34]. Virions enter the midgut via a blood meal and are either subject to decay (*μ*_*v*_) or enter susceptible midgut cells at rate *β*. On infection, virions are produced at rate *γ* and may escape the midgut at rate *ρ* to then circulate in the hemocoel and infect hemocoel cells (Fig 1). To demonstrate the dynamics of virus replication in this system, we ran a single simulation for the equivalent of seven days with arbitrary values of *β* = 10^−8^ h^−1^, *μ*_*v*_ = 0.1 h^−1^, *α* = 10^−3.5^ h^−1^, *ρ* = 0.05 h^−1^.

### Stochastic model and numerical analyses

The change in the numbers of infected midgut cells over time is described by the second equation of Eq 1. Prior to infection (*M*_*c*_ = 0), when virions are introduced (*G*_*v*_ > 0) the event of a midgut cell becoming infected depends entirely on *G*_*v*_*β*(*c*_*max*_) and the rate at which *G*_*v*_ is depleted depends on the virus decay rate (*μ*_*V*_). With these model assumptions, it is therefore the susceptible cell infection rate (*β*) and virus decay rate (*μ*_*v*_) which govern initial infection of midgut cells.

We assume that the rate at which virions infect susceptible cells (*β*) is low such that infection is established in the midgut by only a few virions, despite the presence of 1000s in the blood meal (*G*_*v*_). Under this assumption, it is therefore possible, depending on the values of *β* and *G*_*v*_, by chance, for an infection not to occur. This is supported by observations that infection in the midgut is established from < 15 cells [28–30] and that it is common for not all mosquitoes in a sample to become infected under experimental conditions [19, 20, 22, 24–26].

The deterministic ODE is incapable of capturing this sort of variation and heterogeneity in virus-cell interactions. By introducing stochasticity, particularly forms of demographic stochasticity, offers the potential to capture the between individual variation observed in vector competence experiments where mosquitoes are given blood meals infected with virus. We therefore develop a stochastic version of Eq 1. In this version of the framework, continuous variables are replaced with integers, and rates are replaced by probabilities. There are nine such probabilistic processes in the stochastic framework corresponding to events that occur in the deterministic model outlined in Eq 1 (Table 1).

**Table 1:** Transitions and reaction rates.

Event	Change	Rate
Virion loss from the blood meal	*G*_*v*_ − 1	*μ*_*v*_ *G*_*v*_
Midgut cell infection	*G*_*v*_ − 1, *M*_*c*_ + 1	*βG*_*v*_(*c*_*max*_ − *M*_*c*_)
Spread of infection between midgut cells	*M*_*c*_ + 1	*αM*_*c*_(*c*_*max*_ − *M*_*c*_)
Virion production in midgut	*M*_*v*_ + 1	*γM*_*c*_
Virion decay in midgut	*M*_*v*_ − 1	*μ*_*v*_ *M*_*v*_
Virion escape to hemocoel	*M*_*v*_ − 1, *H*_*v*_ + 1	*ρM*_*v*_
Virion production in hemocoel	*H*_*v*_ + 1	*γH*_*c*_
Virion decay in hemocoel	*H*_*v*_ − 1	*μ*_*v*_ *H*_*v*_
Hemocoel cell infection	*H*_*c*_ + 1	*βH*_*v*_(*h*_*max*_ − *H*_*c*_)

*G*_*v*_ is the number of free virions in the blood meal, *M*_*c*_ is the number of infected midgut cells, *M*_*v*_ is the number of virions in the midgut epithelium, *H*_*v*_ is the number of virions in the hemocoel and *H*_*c*_ the number of infected cells in the hemocoel. Rates involve *μ*_*v*_ which is the virus decay rate, *β* the rate at which virions infect susceptible cells, *c*_*max*_ the total number of midgut cells, *α* the rate at which virions spread between midgut cells, *γ* the rate at which virions are released from infected cells, *ρ* the rate at which virions escape through the basal lamina to the hemocoel and *h*_*max*_ the total number of hemocoel cells.

To simulate the stochastic dynamics, we use the tau-leap version of the Gillespie algorithm [35] (implemented using the adaptivetau package in R). This algorithm is a brute-force method that uses a set of random number generators to determine which process (drawn randomly from all possible processes based on their respective probabilities) occurs in a given small time interval and how many time steps can be aggregated over before the next process occurs.

#### The dose-response of the probability of mosquito midgut infection

We first focus on midgut dynamics and show how a low rate at which virus infects susceptible cells (*β*), relative to the number of input virions, generates stochasticity in the mosquito-virus interaction that can explain the dose-response of the probability of mosquito midgut infection observed in experiments. To do this, for a single set of parameter values, we ran the stochastic model 30 times; simulating viral dynamics in 30 individual mosquitoes. The model was run for 124 hourly time steps. We then determined the proportion of simulated mosquitoes developing a midgut infection as the number of simulations where there was at least one infected midgut cell (*M*_*c*_ > 1) as the numerator and the total number of simulations as the denominator. This was evaluated at the end of the 124 hourly time steps. We repeated this across a range of values for *G*_*v*_ that reflect possible virus concentrations in a blood meal, to explore the possibility of a dose-response. Initial input *G*_*v*_ was between therefore 10^3^ and 10^10^ virus particles per ml, multiplied by 0.003, assuming this is the average size of a mosquito blood meal [36].

As outlined above, the initial virus numbers (*G*_*v*_), the rate at which virus infects susceptible cells (*β*) and the virus decay rate (*μ*_*V*_) influence the probability of midgut infection. We therefore carried out a sensitivity analysis, varying *β* between 10^−14^ and 10^−4^ h^−1^ and *μ*_*V*_ between 1/36 and 1/6 h^−1^ and repeated the stochastic simulations with each value of *Gv* to quantify the effects of these parameters on the probability of mosquito midgut infection as a function of virus dose. The range of *μ*_*v*_ used was chosen to reflect viral decay due to digestion of the blood meal and decay due to other factors [37]. When varying *β*, *μ*_*v*_ was set to 0.02 h^−1^ and when varying *μ*_*v*_, *β* = 10^−8^ h^−1^. Other parameters were kept constant: *γ* = 20, *α* = 10^−4^ h^−1^ and *ρ* = 0.12 h^−1^. To aid visualisation of the simulated dose-response curves, we fitted logistic regression models to the stochastic model outputs, with input virus concentration (*G*_*v*_) the explanatory variable and the proportion of simulations resulting in midgut infection the dependent variable.

#### The effect of stochasticity and input number of virions on dissemination patterns

We next explored the effects of variation in the number of input virions (*G*_*v*_) on virus dynamics across individual mosquitoes that develop a midgut infection and how stochasticity influences dissemination patterns. We acknowledge here that we only account for the time it takes for virus to move from the midgut to the hemocoel and not to salivary glands once dissemination has occurred.

To quantify the effect of the number of input virions on the variation in the time to dissemination; determining some of the duration of the EIP, we ran 30 simulations for each of four different values of *G*_*v*_ for an equivalent of 14 days. Across all simulations, the rate at which virions infect susceptible cells was set to 10^−7^ h^−1^, the virus decay rate (*μ*_*v*_) to 0.1 h^−1^, the virus production rate (*γ*) to 10 h^−1^, the rate at which virions spread between midgut epithelial cells (*α*) was 10^−6^ h^−1^ and the escape rate (*ρ*) was 0.005 h^−1^. On completion of the simulations, we summarised the range of values for *M*_*v*_ across runs at 24 hrs and the range in the time taken for midgut infection to be established. Next, simulations for which *M*_*c*_ remained equal to zero were discarded so that only those with ‘midgut’ infections remained. From these, for each set of simulations corresponding to a different value of *G*_*v*_, we determined the proportion of simulations where *H*_*c*_ > 0, indicating a ‘disseminated’ infection at intervals of 24 time steps (equivalent to per day), taking the last output for each daily time step.

#### Application to vector competence experiments

We demonstrate that the stochastic model can yield similar results to data generated from vector competence experiments. We selected two published studies which used the HND strain of ZIKV to infect *Ae. albopictus* colonised from Long Island, USA and *Ae. aegypti* colonised Poza Rica, Mexico [38, 39]. Combining these two studies provided three different virus concentrations for which to quantify the dose-response for each species in addition to three time points post blood meal for quantifying the probability of dissemination given infection as a function of time (Table 2).

**Table 2:** Data used in model fitting.

Species	Dose	Time	Infected	Disseminated	Total	Reference
*Ae. aegypti*	8.9	21	20	19	22	[38]
*Ae. aegypti*	7.7	21	14	12	30	[38]
*Ae. aegypti*	6.6	21	5	2	30	[38]
*Ae. aegypti*	4.6	21	1	0	30	[38]
*Ae. albopictus*	8.9	21	30	28	30	[38]
*Ae. albopictus*	7.5	21	28	21	30	[38]
*Ae. albopictus*	5.9	21	10	4	30	[38]
*Ae. albopictus*	4.1	21	3	2	30	[38]
*Ae. aegypti*	8.3	4	27	15	30	[39]
*Ae. albopictus*	8.3	4	21	6	30	[39]
*Ae. aegypti*	8.3	7	26	20	30	[39]
*Ae. albopictus*	8.3	7	29	25	30	[39]
*Ae. aegypti*	8.3	14	22	19	30	[39]
*Ae. albopictus*	8.3	14	21	21	30	[39]

Dose is virus concentration in the blood meal provided as log_10_ PFU/ ml. Time is days post ingestion of blood meal that the assay to detect infection or dissemination was carried out. Infected, disseminated and total are numbers of indvidiual mosquitoes. See references for further experimental details. See Fig 6 for graphs of the proportions as a function of dose and time.

Our approach was to first fit the stochastic model of midgut infection dynamics to the data on the proportion of mosquitoes with a midgut infection as a function of virus concentration, to estimate the virus decay rate (*μ*_*v*_) and the rate at which virions infect susceptible cells (*β*). We then fitted the model including midgut and hemocoel dynamics to the data on the proportion of infected mosquitoes with disseminated infection as a function of time, estimating the rate at which virus spreads to new midgut epithelial cells (*α*), the virus production rate from infected cells (*γ*) and the rate at which virus escapes from the basal lamina (*ρ*). We made the simplifying assumption that the virus production rate and rate at which new cells are infected are the same for all cell types in the mosquito.

For model fitting, we used the approach described by Wood [40]. Both the observed data and the simulation outputs were summarised by a statistical model and the coefficients of the statistical model used in a pseudo-likelihood function, assuming that the coefficients follow a multivariate normal distribution:

$$\begin{array}{c} l_{s}\left(\theta \right)=-\frac{1}{2}{\left(s-{\hat{\mu }}_{\theta }\right)}^{T}{\hat{\sum }}_{\theta }^{-1}\left(s-{\hat{\mu }}_{\theta }\right)-\frac{1}{2}log\left|{\hat{\sum }}_{\theta }\right| \end{array}$$ (2)

where *s* is a vector of summary statistics, *θ* represents the unknown model parameters, *μ*_*θ*_ is the unknown mean vector and ∑_*θ*_ the unknown covariance matrix.

We assumed that the data concerning the proportion of mosquitoes with midgut infection and the proportion of mosquitoes with a disseminated infection, given a midgut infection, follow a binomial distribution. The fitting method for midgut infection dynamics proceeded as follows: i) for a given set of parameter values, the model was run 30 times across each of seven input virus values (*G*_*v*_) from 10^5^ to 10^8^ to obtain the proportion of simulations, at each virus dose, that resulted in infection (defined as at least one midgut cell becoming infected, *M*_*c*_ > 0); ii) the simulations across virus concentrations were themselves repeated 30 times; iii) the resulting proportions as a function of *G*_*v*_ were used to fit logistic regression models, to obtain coefficients for the intercept and slope; iv) these coefficients were used to evaluate the likelihood of observing the logistic regression coefficients estimated from the observed data using Eq 2. For the model of midgut and hemocoel dynamics, the process was the same, but using a single value for *G*_*v*_ and fitting logistic regression models to the proportion of simulations where at least one hemocoel cell was infected (*H*_*c*_ > 0) as a function of time. To estimate, in daily time steps, the proportion of simulations for which *H*_*c*_ > 0, we rounded up time steps in model outputs to daily equivalents and took the last run for each ‘day’ to assess whether by that point *H*_*c*_ > 0. Maximum likelihood fitting was implemented using a simulated annealing algorithm [41]. Within the fitting algorithm we constrained the virus production rate (*γ*) to < 1000 h^−1^ and the escape rate (*ρ*) and cell spread (*α*) to be < 1 h^−1^. We first fitted the model to the probability of midgut infection as a function of virus dose for the two species, from [38] to estimate *μ*_*v*_ and *β*. We fitted a single estimate for *μ*_*v*_ between the species but separate values for *β*. For fitting, *ρ* was arbitrarily fixed to 0.05, *γ* to 1 and *α* to 10^−4^. With the fitted values for *μ*_*v*_ and *β*, we then fitted the dissemination model to the proportion of mosquitoes with disseminated infection from [39]. We assumed separate parameter values for *ρ*, *γ* and *α* between the two species. The code for implementing the fitting process is provided in a Github repository.

## Results

**Fig 2:**
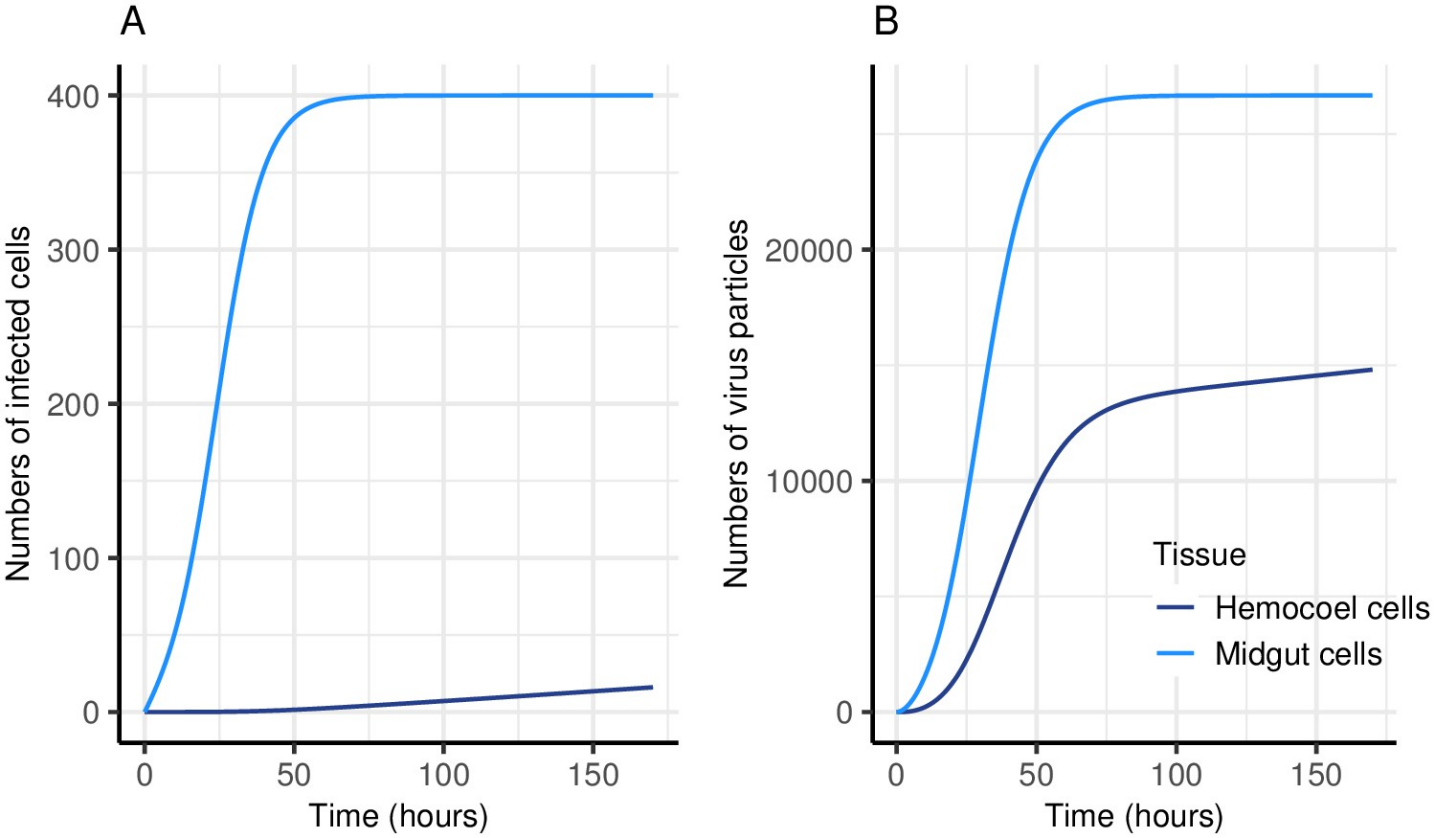
Simulation of virus infection and replication within a single mosquito. A: Numbers of infected cells over time. B: Numbers of virus particles over time. The model tracks numbers of virions in the blood meal (*G*_*v*_), numbers of infected midgut cells (*M*_*c*_) (total cells 400 (*c*_*max*_)), numbers of virions produced in the midgut (*M*_*v*_), numbers of infected hemocoel cells (*H*_*c*_) (total cells available 900 (*h*_*max*_)) and numbers of virions in the hemocoel (*H*_*v*_). The model is run in hourly time steps and a single simulation was run for the equivalent of seven days, with an initial input of 3000 virus particles (*G*_*v*_). The rate at which susceptible cells become infected (*β*) was set to 10^−8^ h^−1^, the virus decay rate (*μ*_*V*_) was 0.1 h^−1^, the rate virions spread between cells (*α*) was 10^−3.5^ h^−1^, the rate at which virions are produced by infected cells (*γ*) was 10 h^−1^ and the rate virions escape from the midgut into the hemocoel (*ρ*) was 0.05 h^−1^.

The ODE model in Eq 1 simulates virus infection and replication dynamics in the midgut and hemocoel of a single mosquito. Including a parameter (*ρ*) for the rate at which virions can escape through the basal lamina into the hemocoel, enables simulation of the delay observed between midgut infection and subsequent dissemination to other mosquito tissues (Fig 2). In our model, as the numbers of midgut and hemocoel cells are limited, the numbers of virus particles produced per unit time eventually plateaus.

The ODE model will always produce the same results given the same parameter values and starting numbers of virions (*G*_*v*_) and is therefore not reflective of experimental infections of mosquitoes with virus where not all mosquitoes become infected and the time it takes for virus to disseminate varies between individuals. Assuming that infection and replication within an individual mosquito is essentially a stochastic process, we show that this can be reflected by a stochastic form of the model in Eq 1, with multiple simulations representing infection in multiple individual mosquitoes.

### Stochastic processes can generate the dose-response of the probability of mosquito midgut infection

**Fig 3:**
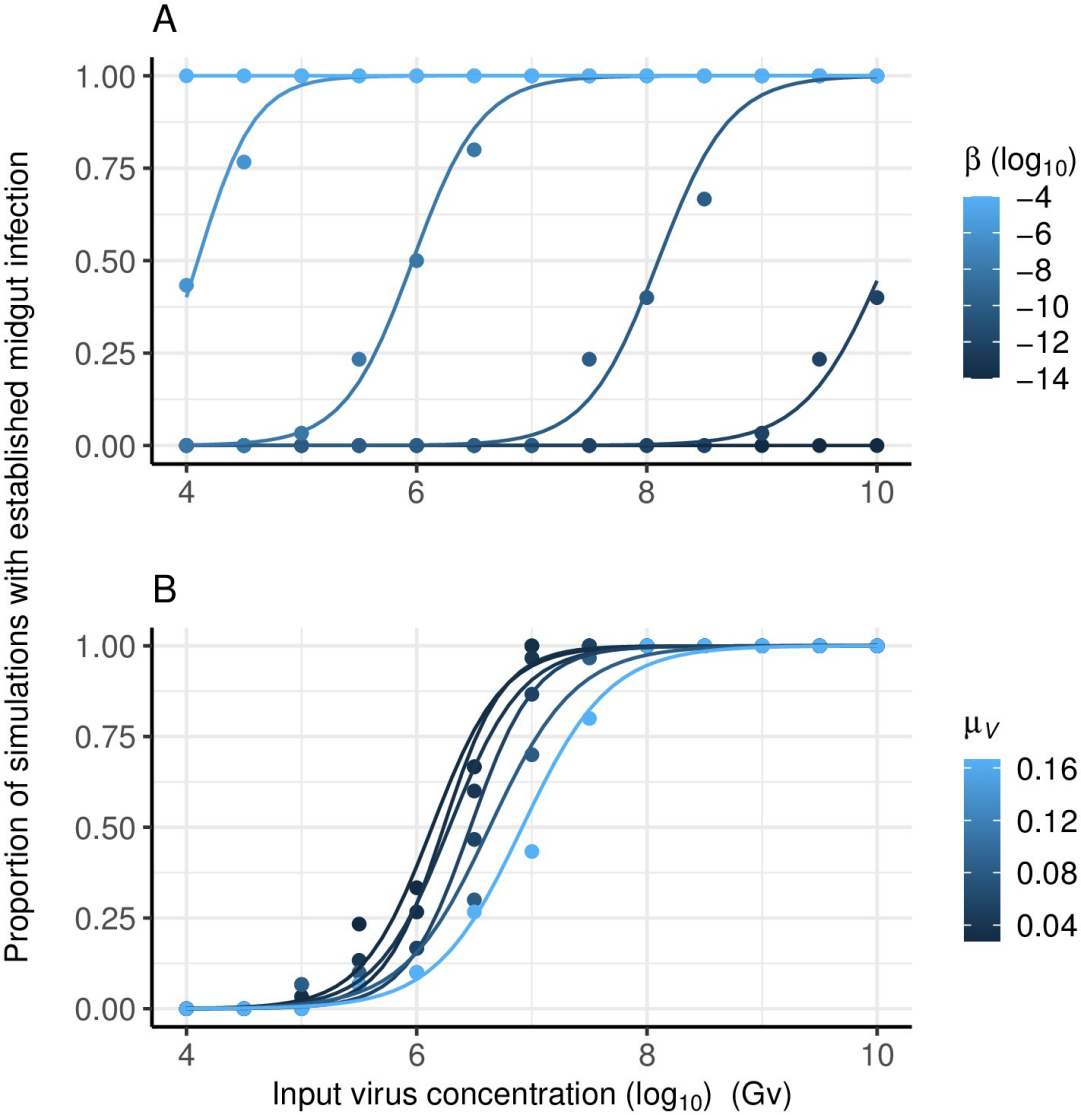
The dose-response of the probability of mosquito midgut infection. Model outputs (points) and fitted logistic regression (lines). Showing the effect of varying: A) the rate at which virions infect susceptible cells (*β*); and B) the virus decay rate (*μ*_*V*_). Each point reflects the results of 30 model simulations. For A, *μ*_*v*_ was set to 0.02 h^−1^, the virus production rate (*γ*) was 20 h^−1^, the rate of cell spread (*α*) was 10^−4^ h^−1^ and the escape rate (*ρ*) 0.12 h^−1^.

A sigmoidal dose-response of the probability of midgut infection (Fig 3) emerges from realizations the stochastic process(es) for increasing numbers of input virions (*G*_*v*_). This dose-response is only apparent when the rate at which a virus infects susceptible cells (*β*) is sufficiently small relative to the input number of virions (*G*_*v*_), otherwise a midgut infection is established with probability one. With respect to sensitivity analysis, lower values of *β* shift the sigmoid curve to the right; the lower the rate the more input virus is required to guarantee all simulations resulted in a midgut infection (Fig 3A). Changes in the rate at which virus is cleared from the midgut (*μ*_*V*_) had less of an effect on the resulting dose-response curve relative to changes in *β* (Fig 3B), over the range of parameter values used.

### The number of virions in the blood meal impacts the duration of the extrinsic incubation period

**Fig 4:**
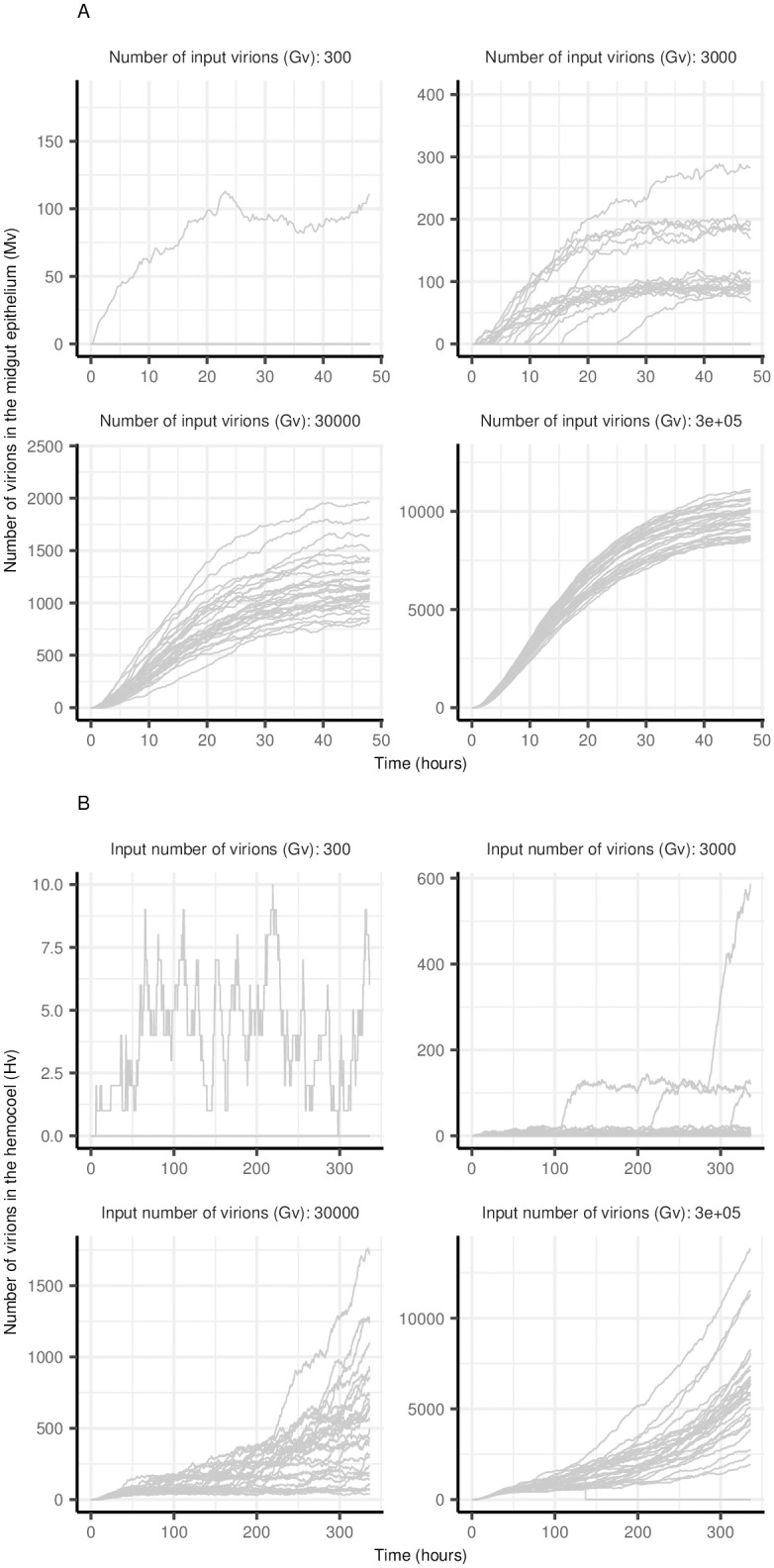
Effect of the input number of virions on viral replication dynamics across tissues. Each line represents a single run of the stochastic model. A: Number of virions in the midgut epithelium (*M*_*v*_) and B: Number of virions in the hemocoel (*H*_*v*_). Parameter values for all simulations were: the rate virions infect susceptible cells, *β* = 10^−7^ h^−1^; virus decay rate, *μ*_*v*_ = 0.1 h^−1^; virus production rate *γ* = 10 h^−1^; rate virions spread between cells, *α* = 10^−6^ h^−1^; and virus escape through the basal lamina, *ρ* = 0.005 h^−1^.

**Fig 5:**
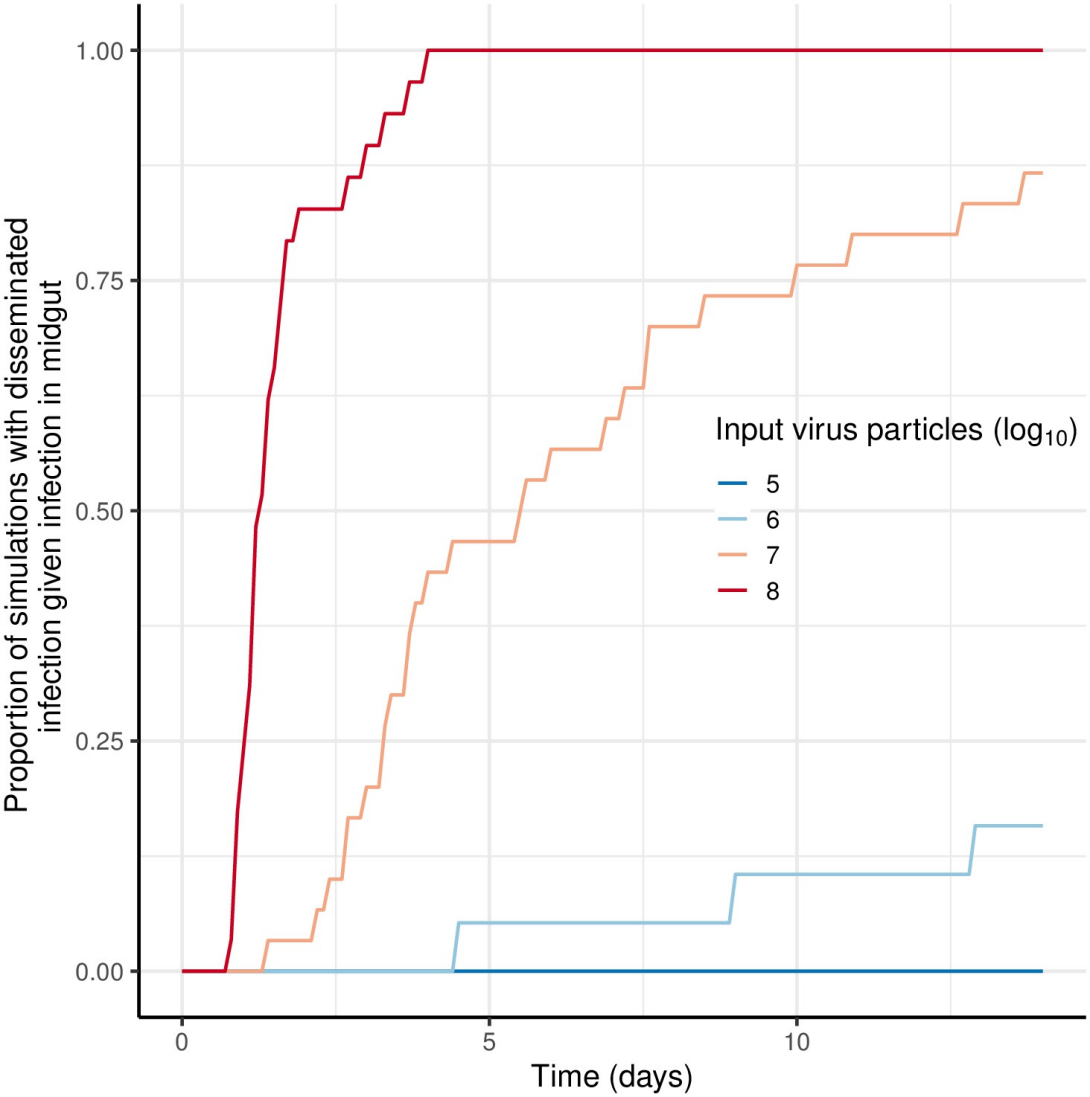
Effect of input number of virions (*G*_*v*_) on probability of dissemination as a function of time. Parameter values for all simulations were: the rate virions infect susceptible cells, *β* = 10^−7^ h^−1^; virus decay rate, *μ*_*v*_ = 0.1 h^−1^; virus production rate *γ* = 10 h^−1^; rate virions spread between cells, *α* = 10^−6^ h^−1^; and virus escape through the basal lamina, *ρ* = 0.005 h^−1^.

When the input number of virions (*G*_*v*_) falls within the range that produces a sigmoid dose-response curve (as shown in Fig 3), the amount of virus in the midgut epithelium (*M*_*v*_) at any given time varies substantially (Fig 4A). For simulations with *β* = 10^−7^ h^−1^ and *G*_*v*_ = 30, 000 (equivalent to a mosquito taking 0.03 μL of a blood meal containing 10^7^ PFU/ml), the number of midgut virions (*M*_*v*_) ranged from *c*. 500 to > 1500 at 24 hrs (Fig 4A, *Gv* = 30, 000). Here, the amount of starting virus (*G*_*v*_) not only impacts whether or not an infection will establish, but also the time since the start of the simulation (post ‘blood meal’) when midgut infection initiates and replication within midgut cells begins; the more virions present the more likely infection will be initiated earlier. For simulations shown in Fig 4A when *G*_*v*_ = 3000, the difference between simulations in the time that midgut infection commenced was *c*. 24 hrs, compared with < 3 hrs for the two higher values of *G*_*v*_ (Fig 4A). This variation in the time the infection begins, and the number of virions produced in the midgut (*M*_*v*_) therefore has implications for the number of virions available to escape to the hemocoel at any given time and, therefore, starting *G*_*v*_ affects the time to dissemination (Fig 4B). An increase in the number of input virions increases the chances of infection starting earlier and thus the chances of earlier dissemination are also increased and therefore a shorter EIP is more likely (Fig 5).

### Model fits to data from vector competence experiments

**Fig 6:**
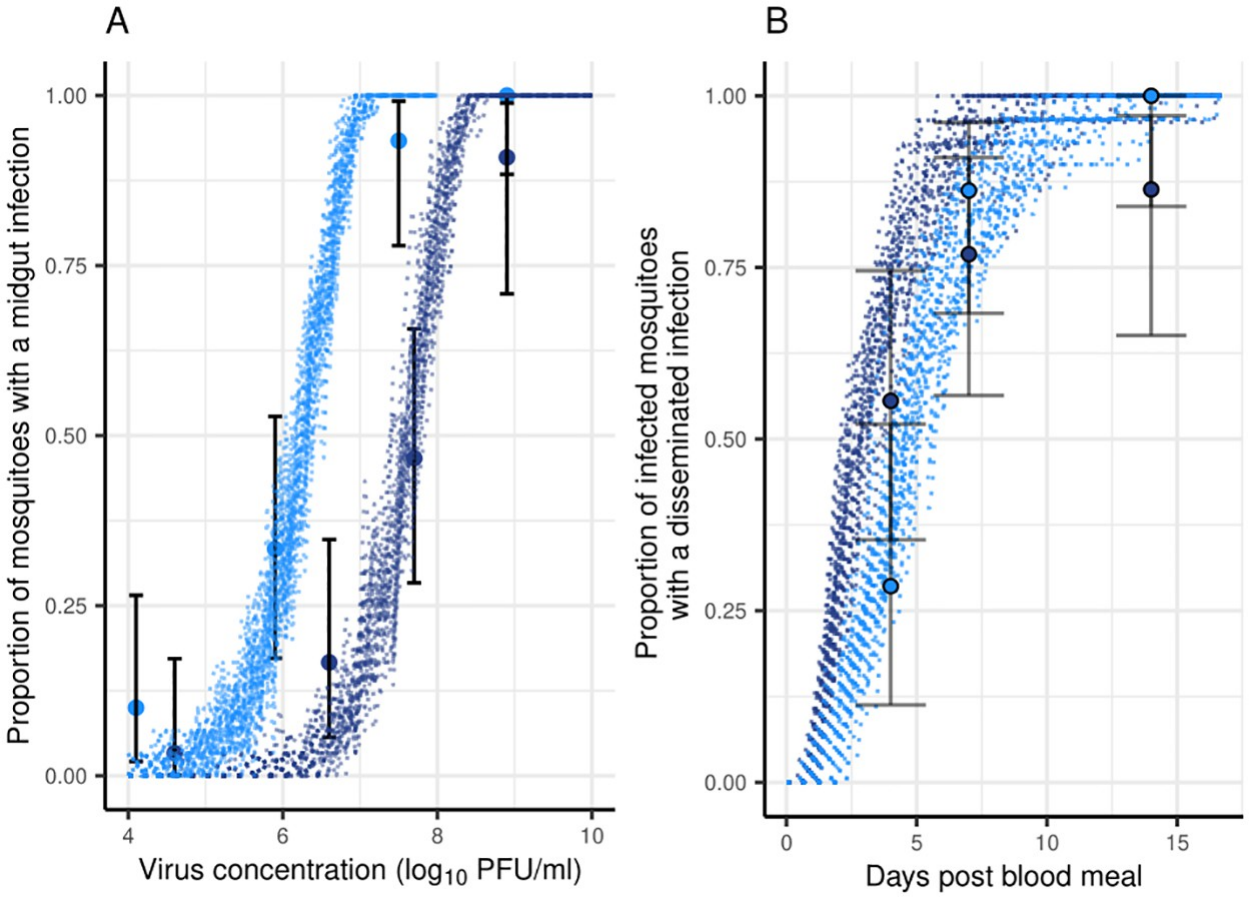
Stochastic model fits to vector competence data for *Ae. aegypti* and *Ae. albopictus* with HND strain of Zika virus. A: dose-response of the probability of midgut infection. B: time-response of the probability of dissemination. Points are observed data from [38, 39] (Table 2) with 95% binomial confidence intervals. Lines represent 30 simulations of ‘model experiments’, each using 30 simulations at each virus dose, using parameter value estimates obtained from maximum likelihood fits to the data. These preliminary fits to the data resulted in the following parameter estimates for *Ae. aegypti*: *μ*_*v*_ = 0.097 h^−1^, *β* = 10^−8.891^ h^−1^, *α* = 10^−3.049^ h^−1^, *γ* = 5.763 h^−1^ and *ρ* = 0.319 h^−1^ and for *Ae. albopictus*: *μ*_*v*_ = 0.097 h^−1^, *β* = 10^−7.520^ h^−1^, *α* = 10^−4.124^ h^−1^, *γ* = 17.429 h^−1^, and *ρ* = 10^−3.000^ h^−1^.

To demonstrate that the model can produce results similar to those observed in vector competence experiments, we fitted the stochastic model to data from experimental infections of *Ae. aegypti* and *Ae. albopictus* with Zika virus strain HND [38, 39], generating stochastic outputs. Preliminary model fits were generally capable of reproducing observed patterns in the data for both infection and dissemination (Fig 6). The estimated higher rate of susceptible cell infection (*β*) for *Ae. albopictus* could explain the greater susceptibility for midgut infection, but this did not result in greater permissiveness for dissemination compared with *Ae. aegypti* (Fig 6).

## Discussion

Arbovirus proliferation within infected hosts can lead to 1000s of virions being imbibed by a mosquito when it takes a blood meal on such a host. However, evidence suggests that very few, perhaps two or three orders of magnitude fewer, virions initiate mosquito midgut infections [28, 29]. That these sorts of infections are established by relatively few virions suggests that virus infection is an intrinsically stochastic process. Using a demographic stochastic approach, we have shown that a stochastic model for midgut and haemoceol infection processes can explain relationships between the amount of virus in a blood meal, the probability an infection occurs and the rate of dissemination to other tissues when infection does occur.

The emergence of a dose-response curve for the probability of mosquito midgut infection as a consequence of a low rate of virion infection of susceptible cells (*β*), is a simple property due to founder effects and stochastic establishment of infections. The interaction between the numbers of virions (*G*_*v*_) present and the rate at which virions infect susceptible cells determines the probability of infection and therefore the dose-response curve. Virus decay (*μ*_*v*_) affects this process indirectly by modulating *G*_*v*_ with time post blood meal. However, the subsequent impact on dissemination patterns of this stochastic infection process is less straightforward. Here, our aim has been to unpack virus dissemination within the mosquito using a mechanistic framework. We show what might initially be viewed as a simple dose dependency relationship, is in fact underpinned by complex non-linear dynamics.

Stochastic processes are often used as representation of an inadequate understanding of a physical and/or biological process. They represent uncertainty in details due to precision and knowledge of key processes. Yet here we conjecture that arbovirus infection in mosquitoes is an inherently random process as it is established by relatively few virions. Intrinsic stochasticity is critically influential in biology [42] such as determining gene expression patterns [43], ecological population growth [44] and quantum biological processes such as electron transport in photosynthesis, and magnetic field effects in bird migration [45]. Viruses are no exception. Through laboratory experimentation and modelling of tomato mosaic virus infection, Miyashita *et. al*. [46] demonstrated that the common finding of small (< 10) founding numbers of viral genomes in establishing between-cell transmission is due to the stochastic behaviour of viral genomes; the probability a genome will get degraded is much higher than the probability it will form a replication complex. They concluded that this stochastic process is advantageous in that it essentially isolates adaptive genomes from defective ones, enabling the rapid selection of *trans* acting genes. They argued their finding also helped to explain why some viruses, including poliovirus, have a high ratio of total virions to those able to infect susceptible cells. Their findings may extend to arboviruses. With respect to arboviruses, the amount of virus present in a blood meal taken from a vertebrate host is constrained by vertebrate host factors. Given this constraint, it should be advantageous for arboviruses to evolve a higher probability of infecting mosquito midgut cells. It could be merely that arboviruses are unable to evolve ways of increasing the rate at which they infect mosquito midgut cells, being constrained by the necessity to infect two different organisms. Alternatively, as found for tomato mosaic virus, the resulting stochasticity could be evolutionarily advantageous.

Our model fits to the Zika vector competence data estimated a very low rate of viral infection of susceptible cells (*β*). This parameter (*β*) combines all the processes of viral cell entry, uncoating, successful replication and encapsidation. One possible explanation is that, like tomato mosaic and poliovirus, individual arbovirus virions may exhibit a very low probability of establishing an initial cellular infection relative to the genome degradation rate inside epithelial midgut cells, and that < 10 cells are initially infected in the midgut may be an extension of this process. This bottleneck may be advantageous in suppressing the replication of defective particles early in infection, as per [46].

Our preliminary work here has shown that we can use a mechanistic description of within-mosquito virus dynamics (the model) and parameterise this from experimental data. This provides a novel way in which to interpret dose- and time-response patterns in the mosquito and highlights how a mechanistic model can produce results similar to that observed in vector competence experiments. A next step would be to develop a robust fitting process that could fully account for uncertainty, permitting hypothesis testing. Additional data including viral titers at different time points may also permit analysis and fitting of more complex models. The mechanistic model comes with a computational burden, therefore it could be employed after logistic regression analyses of the probability of midgut infection, dissemination and salivary gland infection to test for biologically significant differences between experimental treatments [22]. Adding additional transitions from the hemocoel/ tissues to salivary glands would be similar to that for the transition from midgut infection to hemocoel and could be done in future work to extend the model. In addition, our study only models stochasticity arising from a small rate at which virions infect susceptible cells and does not attempt to model the complexities of mosquitoes imbibing blood from a vertebrate host, which may itself introduce further stochasticity.

In our model, the influence of the input virus concentration on the time to dissemination was due to the time of initiation of midgut infection, with later times of establishment leading to a lag in virus accumulation in the midgut and therefore a longer duration before virus finally escaped. This suggests the possibility that mosquitoes taking a blood meal at the peak of host viremia will have a shorter EIP than those taking a blood meal at the beginning and end. Similarly, hosts producing higher viremias will cause not only more mosquitoes to become infected but also for those mosquitoes to experience a shorter EIP. This has been demonstrated under laboratory conditions for Zika virus [21, 22]. While Mayton *et. al*. found that a second blood meal seven days post infection did not shorten the EIP [47], Armstrong *et. al*. [48] found that a second blood meal at three days post infection shortens the EIP due to micro-perforations in the basal lamina. Our model does not account for any change in the rate at which virions escape through the basal lamina (*ρ*) with time post blood meal and with subsequent blood meals. Other model assumptions which could be relaxed in future work include that infected cells can immediately release progeny virions and that there is no cell renewal in the midgut. We do not model salivary gland infection dynamics and we assumed that the rates of virus replication and escape were the same between midgut and hemocoel cells, whereas in reality there could be differences. More complex modelling and detailed data on virus dynamics in the mosquito would be required to estimate these differences and explore mechanisms. Despite these assumptions our model gives insights into the mechanisms driving vector competence and parameters of importance in causing differences between mosquito species, populations and viral strains.

Vector competence experiments often aim to compare the probability of infection as well as dissemination and/ or transmission between viral strains, mosquito species or different treatments using standard statistical tests. Our model fits to data on Zika virus infection in *Ae. aegypti* and *Ae. albopictus* demonstrated that further insights can be gained from fitting mechanistic models. While the data appeared to suggest a similar dissemination rate and thus duration of the EIP between the two species (Fig 6), our model fit suggested that the rate of viral escape from the basal lamina was actually substantially lower for *Ae. albopictus*, despite a lower-dose response of the probability of midgut infection in this species. This has been observed experimentally for dengue virus [24, 49]. Further insights could likely be gained if models were also fitted to data on viral titers over time in addition to proportions of mosquitoes with midgut and disseminated infections. Simulations over multiple input virus concentrations also demonstrated that there is substantial heterogeneity (Fig 5) in dynamics even for concentrations where all mosquitoes become infected and considering this in experimental design when treatments are going to be compared may help to inform sample size allocations across virus concentrations in the blood meal.

Our fitting of the model to ZIKV data is intended as an example of how the model could be used to gain further insight into processes determining results from vector competence experiments. The outputs of this example are specific to ZIKV and *Aedes aegypti*/*Ae. albopictus* and the context within which these studies were carried out, but the model is intended to be extendable to other mosquito-virus systems. As suggested by Chen *et al*. [50], establishing an open-source repository where results from vector competence experiments could be submitted when published would assist the ability for meta-analyses of existing data. When vector competence studies are carried out, we suggest the design of experiments that produce data permitting the quantification of both the dose-response of the probability of mosquito midgut infection and the time-response of the probability of dissemination. As outlined by Wu *et. al*. [51] improved data reporting standards for vector competence experiments would be valuable. Combining improved data reporting standards with mechanistic approaches to understanding and analysing outputs will strengthen conclusions made from vector competence experiments comparing species and virus strains to those assessing novel control approaches.
